# Optimizing Care for the Preterm Infant

**DOI:** 10.3390/children9060778

**Published:** 2022-05-25

**Authors:** Elizabeth Asztalos

**Affiliations:** Department of Newborn and Developmental Paediatrics, Sunnybrook Health Sciences Centre, University of Toronto, Toronto, ON M4N 3M5, Canada; elizabeth.asztalos@sunnybrook.ca

Preterm birth remains an ongoing global health issue with over 15 million infants born preterm annually [1]. However, a significant number of these infants are born less than 28 completed weeks and may experience challenges starting even before the birth, with the birth process, and in the first few weeks after birth. Advances over the last decades have allowed clinicians to provide care to these smaller and immature preterm infants such that even the most fragile infants below 25 weeks gestation have improved survival with the hope and goals of improving neurodevelopmental outcomes at 2–3 years of age and beyond.

The purpose of this Special Issue entitled “Health Care in Premature Infants” is to summarize new information from experts in the field of neonatal care along many fronts. Preterm infants, in particular those born before 28 completed gestation weeks, experience a myriad of complications related to their preterm birth which include severe chronic lung disease, significant and resistant patent ductus arteriosus, major intracranial pathology such as intraventricular hemorrhage and ischemia, infections and even challenges from the beginning in resuscitation following a preterm birth.

Over the last decade, new efforts have been done to explore optimal approaches in the management of the extreme preterm infant in the delivery setting. Schwaberger et al. [2] explore current guidelines and explore challenges currently existing while Law et al. [3] present a pilot feasibility trial which forms the basis of an upcoming large international clinical trial set to start recruitment in 2022.

Cranial hemorrhages in the small preterm infant continue to pose as major consequences and many strategies are explored in attempts to reduce their incidences. Strategies on protecting the brain are explored by Gross et al. [4] and Persad et al. [5].

Of significant importance is who this infant will become and how to support the surviving infant to become a child who is able to function in today’s challenging times and societies around the globe. These are highlighted in different articles in this Special Issue [6,7,8,9].

The care of the small and very vulnerable preterm infant continues to be challenging and requires consistent review and study [9,10,11,12,13,14].

I believe that this issue helps to contribute to consolidating the current body of information with the hopes of enhancing and stimulating further studies on all spectra of care for these vulnerable infants.
